# Analysis of the LRRK2 p.G2019S mutation in Colombian Parkinson's Disease Patients

**Published:** 2015-09-30

**Authors:** Andrés Felipe Duque, Juan Carlos Lopez, Bruno Benitez, Helena Hernandez, Juan José Yunis, William Fernandez, Humberto Arboleda, Gonzalo Arboleda

**Affiliations:** 1 Grupo de Neurociencias. Facultad de Medicina, Universidad Nacional de Colombia, Bogotá, Colombia; 2 Departamento de Patología. Facultad de Medicina, Universidad Nacional de Colombia, Bogotá, Colombia; 3 Departamento de Medicina Interna. Facultad de Medicina, Universidad Nacional de Colombia, Bogotá, Colombia; 4 Departamento de Pediatría. Facultad de Medicina, Universidad Nacional de Colombia, Bogotá, Colombia

**Keywords:** Parkinson´s disease, Dardarin, LRRK2, p.G2019S mutation, Colombia

## Abstract

**Introduction:**

Mutations in the leucine-rich repeat kinase 2 gene (*LRRK2* or Dardarin) are considered to be a common cause of autosomal dominant and sporadic Parkinson´s disease, but the prevalence of these mutations varies among populations.

**Objective:**

to analyzed the frequency of the *LRRK2* p.G2019S mutation (c.6055 G>A transition) in a sample of Colombian patients.

**Methods:**

In the present study we have analyzed the frequency of the *LRRK2* p.G2019S mutation in 154 patients with familial or sporadic Parkinson Disease, including early and late onset patients, and 162 normal controls.

**Results:**

Our results show occurrence of this mutation in two cases (2/154, 1.3%) with classical Parkinson´s signs, and one completely asymptomatic control (1/162, 0.6%).

**Conclusion:**

The p.G2019S mutation is not an important causal factor of Parkinson Disease in Colombia having similar frequencies to those reported in other Latin American populations.

## Abstract (es)

**Introducción:**

Mutaciones en el *LRRK2 *(del inglés gen leucine-rich repeat kinase 2) o Dardarina se consideran una causa común de enfermedad de Parkinson autosómica dominante. Sin embargo, la prevalencia de estas mutaciones varia en diferentes poblaciones.

**Objetivo:**

analizar la frecuencia de la mutación p.G2019S (transición c.6055 G>A) del gen *LRRK2*en una muestra de pacientes colombianos.

**Métodos:**

En el presente estudio analizamos la frecuencia de la mutación en 154 pacientes con enfermedad de Parkinson familiar o esporádica, y 162 controles normales.

**Resultados:**

se determinó la presencia de la mutación en 2 casos de Parkinson (2/154, 1.3%) los cuales presentan los signos clásicos de la enfermedad y en un control completamente asintomático (1/162, 0.6%).

**Conclusión:**

La mutación p.G2019S no es un factor causal importante de la Enfermedad de Parkinson en la población Colombiana, y muestra frecuencias similares a las reportadas en otras poblaciones latinoamericanas.

## Introduction

Parkinson´s disease (PD) is the second most common neurodegenerative disease after Alzheimer´s disease (AD). PD affects all racial groups and has a fairly uniform worldwide distribution 1,2. In Europe, the prevalence of PD is 1.6/100 3, while in Colombia the prevalence is 4.7/1,000 4.

The predominant feature of PD is the progressive slowness of movement (bradikinesia). Patients initially experience resting tremor, difficulty in the initiation of the movements in the absence of paralysis (akinesia), and muscular rigidity, finally leading to postural instability 5. A subset of patients also develops dementia 6,7. The main pathological features of PD are the selective and progressive loss of dopaminergic neurons in the substantia nigra compact zone of the midbrain, degeneration of the nigrostriatal system and the presence of Lewy bodies 8. 

The contribution of genetic factors to the development of PD has been largely underestimated for many yrs, although as many as 15% of PD cases have a family history of the disease 9. With the recent description of mutations in several genes associated with early-onset autosomal dominant and recessive familial Parkinsonism, it has been suggested that PD can have a genetic basis 10,11. However, the extent of the genetic contribution and the interaction between environmental and genetic factors remain unclear 12.

To date, mutations in several genes have been associated to PD: PARK 1 to PARK 15. Some of these mutations are linked to familial autosomal dominant (PARK 1/4: *alpha-synuclein*, PARK 8: *LRRK2*) or recessive (PARK 2: *Parkin*, PARK 6: *DJ-1* and PARK 7: *PINK1*, PARK 13: *ATP13A2*) forms of PD 10,13,14. 

Recently, diverse studies in different populations have identified mutations in leucine-rich repeat kinase 2 gene (PARK 8: *LRRK2* or Dardarin) in autosomal dominant and sporadic forms of PD 15,16. *LRRK2* mutations cause PD in many different populations and their prevalence in familial and sporadic cases varies across these groups 17,18. Studies of the frequency of *LRRK2* mutations in Latin American countries are scant. In Brazil the prevalence of the *LRRK2* p.G2019S mutation is about 2%, being more prevalent among familial than in sporadic PD cases (8.7% vs 0.76%) 19. In Chilean population the prevalence of this mutation is around 3% 20.

In the present paper we have analyzed the prevalence of the *LRRK2* p.G2019S mutation in a sample of unrelated Caucasian-mestizo PD patients from Colombia. 

## Materials and Methods

We analyzed the frequency of the p.G2019S mutation (c.6055 G>A transition) in a case-control study of 154 PD patients and 162 controls. PD cases were 92 males and 62 females (mean age: 59.5 yrs; SD: 15.0); controls were 60 males and 102 females (mean age: 74.9 yrs; SD: 12.3). Twenty eight patients had early onset PD (less than 40 yrs old: mean age at onset: 29.4 yrs; SD: 7.7). There were 126 late onset PD cases (mean age at onset: 58.2 yrs; SD: 10.5). 130 cases were sporadic (mean onset age: 53.4 yrs; SD: 14.8) and 24 were familial cases (mean onset age: 48.7 yrs; SD: 16.6). Patients and controls were analyzed by an interdisciplinary group at the Movement Disorders Clinic of the National University of Colombia from 2005 to 2014. Diagnosis was done following the United Kingdom Parkinson's Disease Society Brain Bank diagnostic criteria (UK PDSBB) 21. Controls were free of any motor or cognitive alterations and did not have family history of PD. The research protocol was approved by the ethics committee of the Faculty of Medicine, National University of Colombia and both patients and controls signed informed consent authorizing their participation in this study.

DNA was extracted from peripheral blood with the Wizard Genomics DNA isolation Kit (Promega Corporation, Madison WI). *LRRK2* mutation was genotyped following the methodology described by Nichols 16. Genomic DNA was amplified using forward (5´ -TTTTGATGCTTGACATAGTGGAC-3') and reverse (5´-CACATCTGAGGTCAGTGGTTATC-3') primers, in an amplification reaction containing 50 ng of DNA, 25 pmol of each primer, 250 (M of each dNTP, 1.5 mM of MgCl_2_, 2.5 (L of reaction buffer 10X and 1U of Taq polymerase, in a final volume of 25 (L. The amplification conditions were the following: an initial denaturing step at 94º C for 5 min followed by 35 cycles of 94º C for 30 s, 60º C for 30 s, 72º C for 45 s, and a final extension of 10 min at 72º C. The PCR products were then subjected to restriction endonuclease digestion analysis using *Sfc1* enzyme (New England BioLabs, USA), resolved in a 70% NuSieve and 30% SeaKeam agarose gel electrophoresis and visualized with ethidium bromide under ultraviolet illumination. The amplified fragments were of 228 and 101 bp for non-mutated, and 228, 207, 101 and 21 bp fragments for the mutant heterozygotes. Sequencing was done using BigDye terminator following manufacturer's recommendations and analysed by an ABI-PRISM 3500 automatic sequencer (Applied Biosystems, USA). NovoSNP software was used to reveal the mutations.

## Results

The frequency of *LRRK2* p.G2019S mutation was 1.3% in patients (2/154) and 0.6% in controls (1/162), *p* value= 0.9777. The two *LRRK2* p.G2019S mutation cases were heterozygotes for the mutation and exhibited signs of classical PD (Table 1). They both had good response to L-Dopa and experienced L-Dopa induced dyskinesias. The control with the *LRRK2 *p.G2019S mutation has neither motor alteration nor cognitive impairment at the age of 77 yrs, and had no family history of PD.

**Table 1.** Clinical features of the LRRK2 G2019S positive cases.

Features	Patient 1	Patient 2	Control
Sex	Female	Male	Male
Ethnic origin	CM	CM	CM
Family history	no	yes*	no
Age at exam (yrs)	79	67	77
Age at onset (yrs)	71	60	n/a
Bradykinesia	yes	yes	no
Resting Tremor	yes	no	no
Rigidity	yes	yes	no
Postural Instability	yes	yes	no
Asymmetry at onset	yes	yes	no
Dystonia	no	no	no
Behavioral alterations	no	no	no
L-DOPA response	yes	yes	n/a
Cognitive decline	no	no	no
L-DOPA induced dyskinesia	yes	yes	n/a

CM= caucasian mestizo

*affected both mother and half-brother

n/a= non applicable

**Figure 1.**
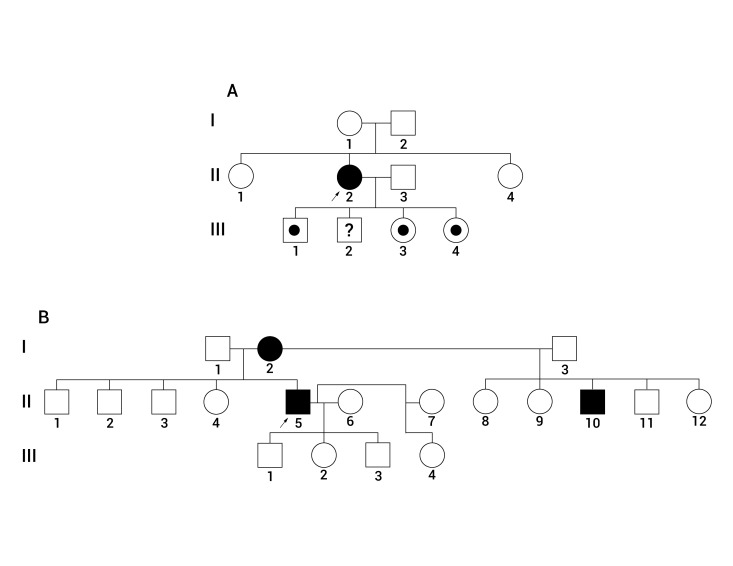
Pedigrees of the patients positive for the LRRK2 pG2019S mutation. **A.** Pedigree of the female patient in which family history was not reported. There is one affected woman (Generation II, individual 2). **B**. Pedigree of the male patient with family history of PD. There are multiple affected individuals (Generation I, individual 2; Generation II, individuals 5 and 10). Open square or circle: Unaffected male or female, filled square or circle: affected male or female, dot inside square or circle: asymptomatic carrier. Pedigrees were drawn using the online freely available Pelican software.

**Figure 2.**
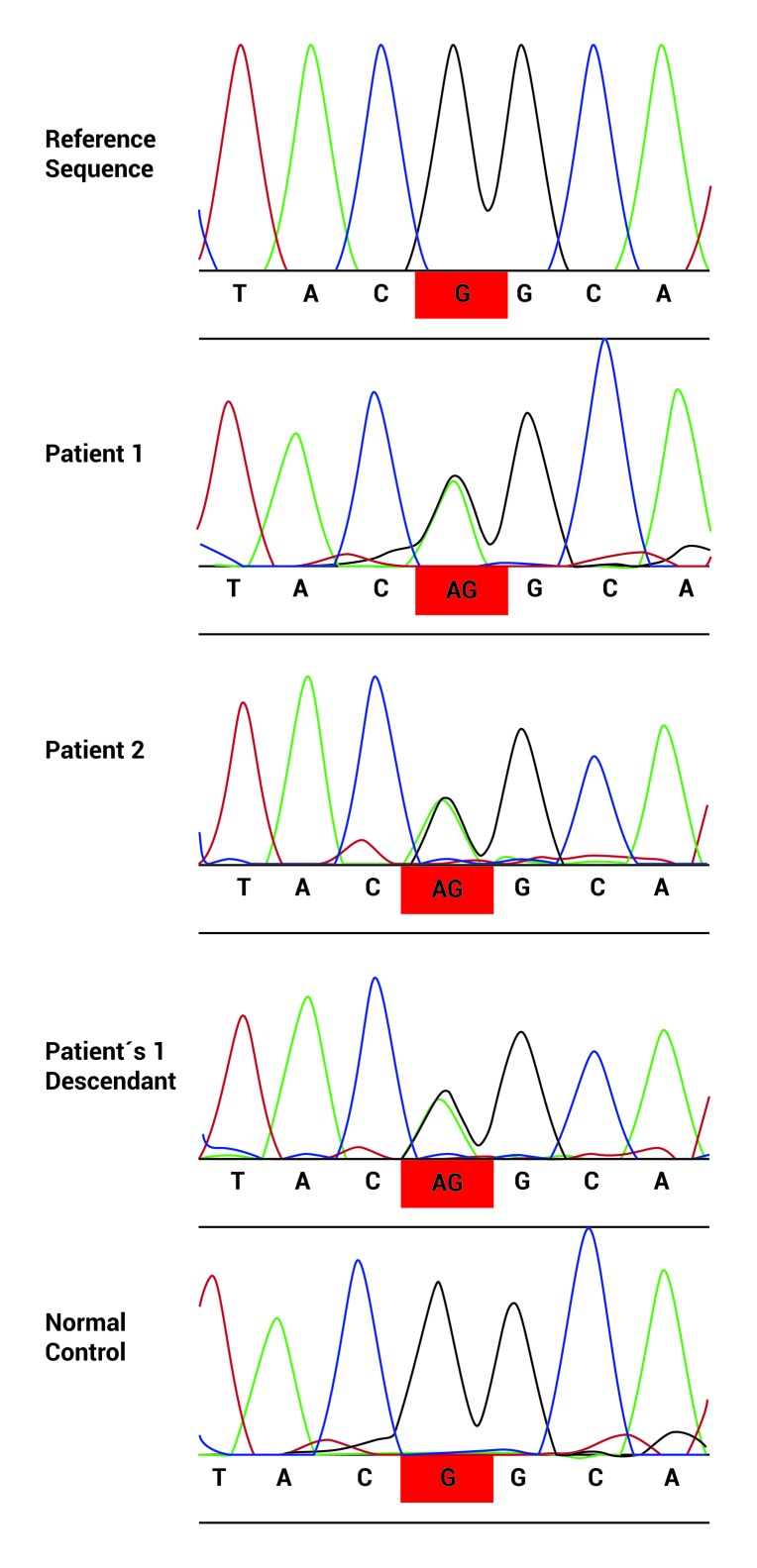
Sequence of the LRRK2 gene that confirmed the LRRK2 pG.2019S mutation in patients 1 and 2, and descendants of patient 1 (It is only shown one of them, but mutation was present in 3 out of 4 descendants).

Family study of one of the positive patients for the *LRRK2* p.G2019S mutation (without family history for the disease) revealed the presence of the mutation in 3 out of 4 of her descendants, none of them have been affected by the disease taking into account that it has been described an age-dependent penetrance of the *LRRK2* p.G2019S and the oldest of them was 42 yrs old at the moment of the examination, followed by a male aged 40, a woman aged 39, and the youngest aged 36. All of them are heterozygotes (Figs. 1A and 2). The family of the other *LRRK2* p.G2019S positive patient, who had family history of PD, refused to participate in the study (Fig. 1B). The control positive for the mutation had no descendants, neither brothers. 

Although the *LRRK2* pG2019S mutation is an infrequent causal factor for PD among Colombian population, there is a tendency for increased risk (OR= 2.118, CI 95%= 0.190-23.616), that is greatly underpowered to consider it significant. 

## Discussion

The etiology of PD is complex and has not been completely clarified 10,13 Mutations in *LRRK2* have been described in different populations, with variation in its frequency. The most common of the *LRRK2* mutations is the p.G2019S. The frequency of this mutation is high among North Africans (41%) 18, followed by Ashkenazi Jews (18.3%) 22, in Europe and North America is around 0.8-6% 23-25, while is lower among Latin Americans 19,20,26 and absent in Asians 27 (Table 2). The most plausible explanation for the observed differences in frequencies is the inherent genetic background of each sample, being Latin-America and Colombia in particular a highly mix population 28, that defines their specific susceptibility to the development of the disease. In fact, the mutation has been traced among different populations and the findings raise the possibility of a common ancestor for this mutation, following what might be considered a demographic and geographic pattern of evolution 29. 

**Table 2.** Frequency of the LRRK2 G2019S in diverse populations around the world.

Population	p.G2019S Frequency (%)	Reference
Chile	5/166 (3.0)	Perez-Pastene et al.^20^
Brazil	3/154 (2.0)	Pimentel et al.^19^
Brazil	3/83 (3.5)	Munhoz et al.^26^
Spain	16/302 (5.3)	Gaig et al.^31^
Spain	8/105 (7.6)	Infante et al.^32^
Spain	5/225 (2.7)	Mata et al.^33^
Italy	5/513 (0.8)	De Rosa et al.^25^
Uruguay and Peru	Uruguay 5/125 (0.04); Peru 1/240 (0.4)	Mata et al.^29^
Argentina	3/55 (5.5)	Gatto et al.^30^
North African Arabs	4/17 (41.0)	Lesage et al.^18^
Azkenashi Jews	22/120 (18.3)	Ozelius et al.^22^
Asian populations	0/675 (0.0)	Tan et al.^27^
Colombia	2/154 (1.3)	Present study

The frequency of the *LRRK2* p.G2019S mutation in the Colombian population is one of the lowest reported in Latin American populations and is one of the lowest in the world. Reports from Peru, Chile and Brazil have described frequencies of this mutation between 0.4-3.5% 19,20,30, and higher frequency in Argentina and Uruguay (4-5%) 30,31. However, the small differences observed in the frequency of this mutation between the present study and other Latin American and European populations studied are more probably related to methodological issues such as sample size and composition (number of familiar and sporadic cases in each study).

Clinical characteristics of the positive *LRRK2* p.G2019S patients do not differ from any other PD cases, coinciding with the phenotype described for *LRRK2* p.G2019S carriers in other reports 15,32-34. The control positive for the mutation was completely asymptomatic at the age of 77 yrs. No motor alteration, no cognitive decline and no signs of any neurological disease were found. This could be explained by the incomplete penetrance of the mutation as previously described for carriers of the *LRRK2* pG2019S over the age of 70 yrs 15,32. 

## Conclusion

Our results demonstrate that the *LRRK2* p.G2019S mutation is an infrequent cause of PD in the present sample, having similar frequencies to other Latin American populations. 
